# Differences of clinical phenotype between familial and sporadic Crohn’s disease in East China

**DOI:** 10.1007/s00384-024-04688-7

**Published:** 2024-07-13

**Authors:** Siyuan Dong, Xiaoxia Xiang, Yu Zhang, Rongbei Liu, Lingna Ye, Qian Cao

**Affiliations:** 1 Department of Gastroenterology, College of Medicine, Sir Run Run Shaw Hospital, Zhejiang University, Hangzhou, 310016 China; 2 Inflammatory Bowel Disease Center, College of Medicine, Sir Run Run Shaw Hospital, Zhejiang University, Hangzhou, 310016 China; 3 Institute of Gastroenterology, Zhejiang University, Zhejiang Province, Hangzhou, 310016 China; 4 Department of Gastroenterology, Haiyan People’s Hospital, Jiaxing, 314300 China

**Keywords:** Crohn’s disease, Familial, Sporadic, Phenotype

## Abstract

**Purpose:**

Family history is one of the strongest risk factors for inflammatory bowel diseases (IBD) while studies about the clinical phenotype of familial IBD are limited. This study aimed to compare the phenotypic features of familial Crohn’s disease (CD) with sporadic CD.

**Methods:**

Familial CD was defined as CD patients having one or more first, second, third, fourth degree, or above relatives with CD. Sporadic CD patients hospitalized during the same period were matched 1:3 by age and gender. Differences in clinical characteristics, phenotype distribution, extraintestinal manifestations, and complications at diagnosis, as well as treatment regimen and surgery, were compared between familial and sporadic CD.

**Results:**

The familial CD was associated with a higher rate of past appendectomy history (*P* = 0.009), more intestinal perforation at onset (*P* = 0.012), more MRI results of anal lesion (*P* = 0.023), and gastrointestinal perforation (*P* = 0.040) at diagnosis, higher rate of past intestinal surgery history (*P* = 0.007), more number of intestinal surgeries (*P* = 0.037), longer duration of follow-up (*P* = 0.017), lower rate of taking biologicals for current maintenance (*P* = 0.043), lower tendency to upgrade to biologicals during follow-up (*P* = 0.013), higher possibility to experience gastrointestinal obstruction (*P* = 0.047), and abdominal abscess during follow-up (*P* = 0.045).

**Conclusion:**

Familial CD is associated with a more aggressive clinical phenotype.

## Introduction

Inflammatory bowel diseases (IBD), including Crohn’s disease (CD) and ulcerative colitis (UC), are chronic and relapsing diseases that distress millions of people worldwide. During the last two decades, the number of IBD patients in Asia including China increased rapidly, attracting more and more attention from doctors [1]. The pathogenic mechanisms behind IBD are not yet fully defined, while the current hypothesis suggests that genetic variations, immunological alterations, shifts in the gut microbiome, and external environmental influences altogether affect disease development [2, 3]. Family history is one of the strongest risk factors for IBD [4–8].

Epidemiological surveys showed that there were great differences in the incidence of IBD among different ethnic groups. The incidence of IBD was the highest among Caucasians, followed by African Americans, and the lowest among Asians. A positive family history increases the risk 10–15 times for IBD development [5, 9, 10]. So we believe that genetic predisposition is a strong factor influencing the development of IBD.

Asian IBDs have shown great differences from Western IBDs, including the genetic factor. Polymorphisms in nucleotide-binding oligomerization domain 2 (NOD2) contribute the largest fraction of genetic risk for Western Crohn’s disease so far, while we found no mutation in the Chinese Han population [11]. A study from Denmark showed that up to 12% of all IBD cases have a positive family history [12]. An American study in 1996 reported the prevalence of familial IBD ranged between 10 and 25% [13]. While in Asians, we used to think that IBD is sporadic. As the incidence rises, family history is becoming more evident. The IBD patients with a family history take 1 to 6% proportions in Asian populations [5, 14–18].

Although many studies have confirmed the influence of family history on the risk of IBD disease, few studies have focused on whether family history influences the clinical features, disease course, and severity of patients with IBD. There is still controversy about the difference between sporadic and familial IBD. Some studies suggest that familial IBD has an earlier onset, higher disease severity, and is more aggressive [12, 19, 20], while others suggest that genetic influences are overestimated and do not show significant differences in clinical features between familial and sporadic IBD cases [7, 15, 21]. There is a lack of studies on familial IBD in Chinese populations, and it is worth exploring whether there are differences in the clinical features between familial and sporadic IBD. Moreover, most familial IBD studies have limited and ambiguous data. Consequently, we performed this study and obtained more accurate information by telephone follow-up. We aimed to provide a better understanding of the effect of familial CD on the clinical phenotype and disease course, which might be helpful for clinical decision-making.

## Materials and methods

### Study design

This hospital-based prospective study was performed at the Department of Gastroenterology, the Sir Run Run Shaw Hospital, Zhejiang University School of Medicine (Hangzhou, Zhejiang Province, China). The study protocol was approved by the Ethical Review Committee of the Sir Run Run Shaw Hospital (no. 20211103–36). Informed consent was obtained from each patient included in the study. The study protocol conformed to the ethical guidelines of the 1975 Declaration of Helsinki (6th revision, 2008) as reflected in a priori approval by the institution’s human research committee.

### Study population

This study included patients recruited in a prospective registry, which has been maintained since 2019.01.01 at the Sir Run Run Shaw Hospital and other hospitals. All adult patients aged 18 years and older with a confirmed diagnosis of CD, UC, or IBD-unspecified (IBDU), seeking care at the Inflammatory Bowel Disease Center of Sir Run Run Shaw Hospital, were eligible for inclusion in the cohort. All patients were followed up and data in this study ended at 2021.12.31.

A family history of IBD was assessed by a detailed questionnaire ascertaining the presence of either CD or UC in a first degree (parent, child, sibling), second degree (grandparent, uncle, aunt), or distant relative. Patients were considered to have familial IBD in the presence of CD or UC in any relative. Patients with no reported family history were considered to have sporadic IBD [4].

### Data collection

Demographics and clinical characteristics including age, gender, growing environment before 14 years old, history of smoking, education, past medical history, breastfeeding time, age at onset, clinical characteristics at onset, course of disease, age at diagnosis, Montreal classification, perianal lesions, MRI results of anal, gastrointestinal tumor, gastrointestinal bleeding, gastrointestinal perforation, gastrointestinal obstruction, abdominal abscess, intestinal fistula, extraintestinal manifestations, age at the first intestinal surgery, resection of the intestine segments > 1 m, time between multiple surgeries, number of surgeries within 1 year of onset, number of surgeries within 2 year of onset, number of intestinal surgeries, history of intestinal surgery, causes of intestinal surgery, number of perianal surgeries, history of perianal surgery, causes of perianal surgery, number of endoscopic surgeries, history of endoscopic surgery, causes of endoscopic surgery, history of appendix surgery, duration of follow-up, current maintenance drug, upgrade to biologicalsals, type of biologicalsals, loss of response to biologicalsals, current disease location, current disease behavior, gastrointestinal tumor during follow-up, gastrointestinal bleeding during follow-up, gastrointestinal perforation during follow-up, gastrointestinal obstruction during follow-up, abdominal abscess during follow-up, intestinal fistula during follow-up were obtained and confirmed.

### Statistical analysis

Continuous variables are presented as medians with interquartile ranges (IQRs), and categorical variables are presented as numbers with percentages. Two-tailed unpaired *t*-test or Mann–Whitney *U* test was used to compare continuous variables, and the χ^2^ test or the Fisher exact test was used to compare categorical variables, as appropriate. The cumulative rate of upgrade to biologicals was compared by survival analysis using the log-rank test. A *P* value < 0.05 was considered statistically significant. Statistical analysis was performed using R software version 4.2.

## Results

### Prevalence of familial IBD

**Fig. 1:**
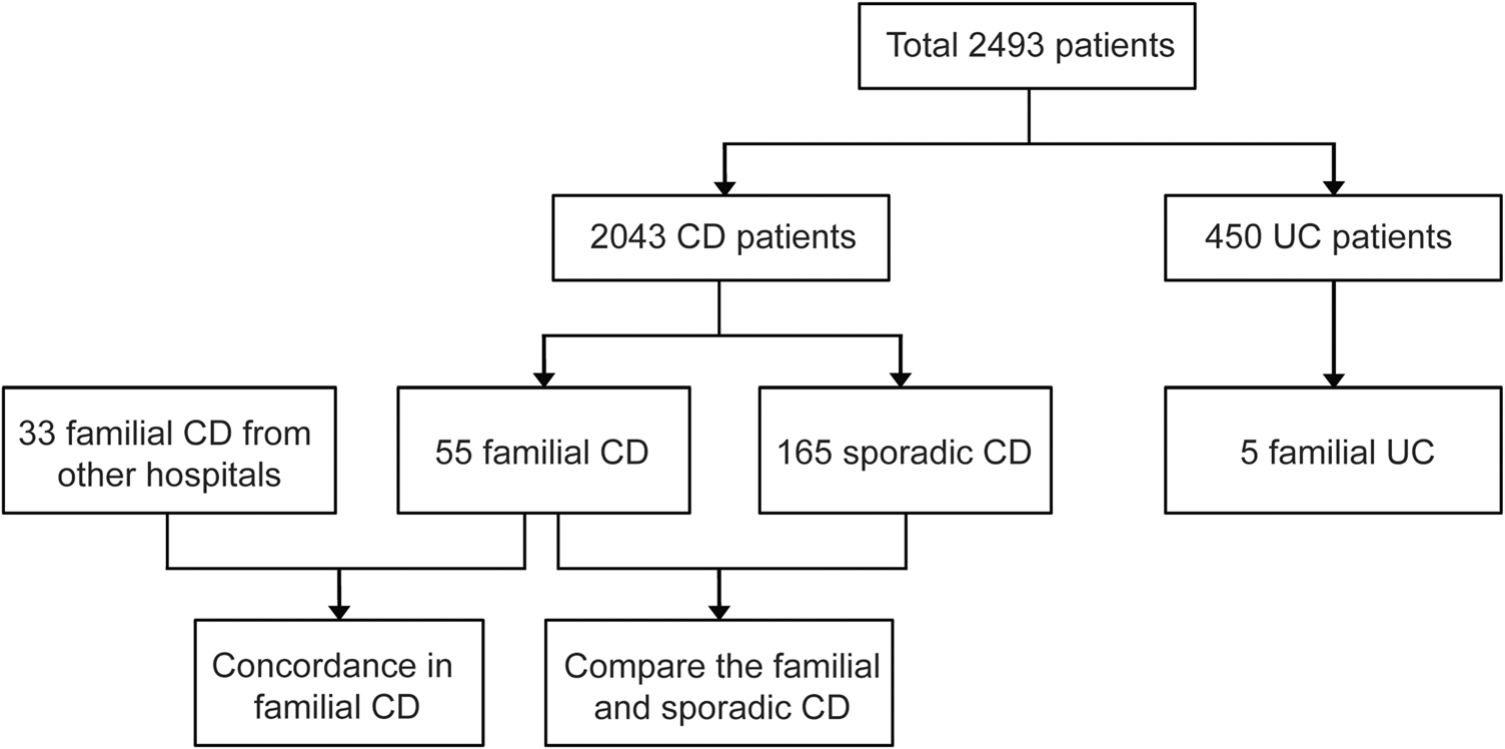
Flow diagram showing the distribution of familial and sporadic IBD

**Fig. 2:**
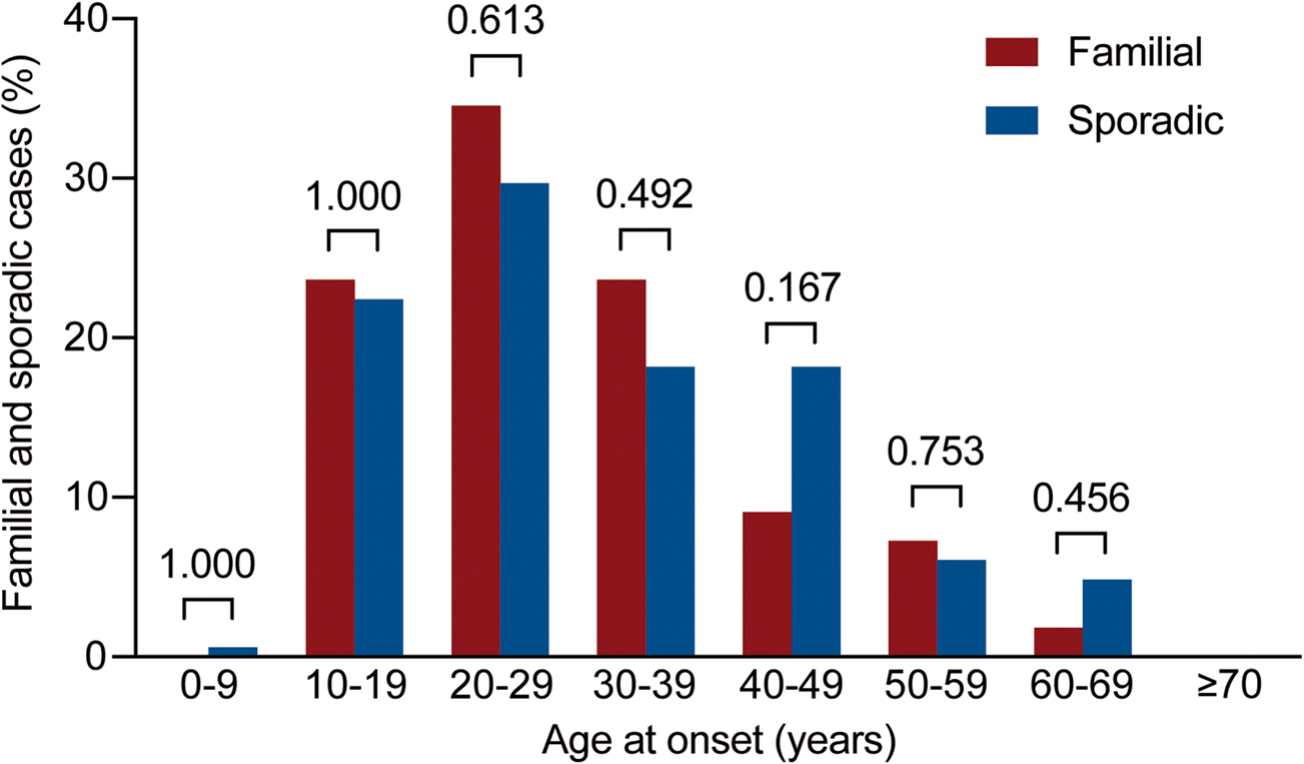
Bar diagram showing familial and sporadic Crohn’s disease (CD) cases by age at onset

At the time of this study, 2493 IBD patients were included, with 2043 CD and 450 UC patients. Overall, 60 out of 2493 IBD patients (2.39%) had familial IBD, including 55 CD patients and 5 UC patients (Fig. 1). The prevalence of family history according to the age of onset is shown in Fig. 2. The number of cases for both familial and sporadic CD increased before 30 years of age with age and decreased after 30 years of age with age. The maximum number of cases for both familial and sporadic CD were in the 20–29-year age group.

### Demographic characteristics of familial CD and sporadic CD

Familial CD and sporadic CD were similar in age, gender, growing environment, history of smoking, education, and breastfeeding time, the comparison did not show significant differences. However, familial CD was associated with a higher percentage of past appendectomy history (20.00% vs 6.67%, *P* = 0.009) (Table 1).

**Table 1:** Demographic characteristics of familial and sporadic Crohn’s disease (CD)

	Familial (*N* = 55)	Sporadic (*N* = 165)	*P* value
Age, years (median [IQR])	35.00 [26.00; 48.50]	35.00 [26.00; 50.00]	0.982
Gender, *n* (%)			0.937
Male	32 (58.18)	93 (56.36)	
Female	23 (41.82)	72 (43.64)	
Growing environment before 14 years old, *n* (%)			
City	6 (10.91)	37 (24.03)	0.061
Town	12 (21.82)	19 (12.34)	0.140
Country	36 (65.45)	98 (63.64)	0.938
History of smoking, *n* (%)			
No	30 (54.55)	83 (53.55)	1.000
Yes	4 (7.27)	15 (9.68)	0.786
Former smoker	7 (12.73)	20 (12.90)	1.000
Passive smoker	14 (25.45)	37 (23.87)	0.958
Education, *n* (%)			
Illiteracy	1 (1.82)	2 (1.30)	1.000
Primary school	6 (10.91)	15 (9.74)	1.000
Junior high school	12 (21.82)	37 (24.03)	0.884
Senior high school	11 (20.00)	25 (16.23)	0.669
Technical college	10 (18.18)	26 (16.88)	0.991
University	14 (25.45)	45 (29.22)	0.720
Master	0 (0.00)	4 (2.60)	0.575
Doctor	1 (1.82)	0 (0.00)	0.263
Past medical history, *n* (%)			
No	39 (70.91)	123 (74.55)	0.724
Appendectomy	11 (20.00)	11 (6.67)	0.009*
Tonsillectomy	1 (1.82)	2 (1.21)	1.000
Infectious mononucleosis	0 (0.00)	0(0.00)	
Eczema	5 (9.09)	16 (9.70)	1.000
Asthma	2 (3.64)	1 (0.61)	0.155
Other autoimmune disorders	1 (1.82)	4 (2.42)	1.000
Breastfeeding time, *n* (%)			
No	7 (12.73)	9 (5.45)	0.129
< 3 months	0 (0.00)	4 (2.42)	0.574
3–6 months	3 (5.45)	10 (6.06)	1.000
6–12 months	19 (34.55)	43 (26.06)	0.299
≥ 12 months	26 (47.27)	76 (46.06)	1.000

*Statistically significant results

Abbreviations; *IQR* interquartile range

### Clinical characteristics at onset and diagnosis of familial CD and sporadic CD

Most clinical characteristics at onset were not significantly different between familial CD and sporadic CD, while intestinal perforation was more common in familial CD (12.73% vs 3.03%, *P* = 0.012) (Table 2).

**Table 2:** Clinical characteristics at onset of familial and sporadic Crohn’s disease (CD)

	Familial (*N* = 55)	Sporadic (*N* = 165)	*P* value
Age at onset, years (median [IQR])	27.00 [20.00; 37.00]	29.00 [21.00; 41.00]	0.436
Clinical characteristics at onset, *n* (%)			
Abdominal pain	32 (58.18)	108 (65.45)	0.418
Diarrhea	22 (40.00)	74 (44.85)	0.638
Bloody stool	9 (16.36)	25 (15.15)	1.000
Abdominal mass	0 (0.00)	1 (0.61)	1.000
Anemia	0 (0.00)	4 (2.42)	0.574
Fever	6 (10.91)	11 (6.67)	0.380
Weight loss	19 (34.55)	42 (25.45)	0.258
Extraintestinal manifestations at onset	3 (5.45)	19 (11.52)	0.299
Perianal lesions at onset	16 (29.09)	45 (27.27)	0.931
Intestinal obstruction	5 (9.09)	21 (12.73)	0.630
Intestinal perforation	7 (12.73)	5 (3.03)	0.012*

*Statistically significant results

Abbreviations: *IQR*, interquartile range

As for clinical characteristics at diagnosis, MRI results of anal lesion (85.45% vs 68.48%, *P* = 0.023) and gastrointestinal perforation (14.55% vs 5.45%, *P* = 0.040) were more common in familial CD. There was no difference in the course of the disease, age at diagnosis, Montreal classification (age, location, and behavior), perianal lesions, gastrointestinal tumor, gastrointestinal bleeding, gastrointestinal obstruction, abdominal abscess, and intestinal fistula (Table 3).

**Table 3:** Clinical characteristics at diagnosis of familial and sporadic Crohn’s disease (CD)

	Familial (*N* = 55)	Sporadic (*N* = 165)	*P* value
Course of disease, years (median [IQR])	6.00 [2.00; 21.50]	11.00 [3.00; 28.00]	0.142
Age at diagnosis, years (median [IQR])	29.00 [21.50; 38.00]	31.00 [23.00; 44.00]	0.358
Montreal classification age at diagnosis, *n* (%)			
≤ 16 years old	4 (7.27)	12 (7.27)	1.000
17–40 years old	39 (70.91)	99 (60.00)	0.198
> 40 years old	12 (21.82)	54 (32.73)	0.174
Montreal classification-location, *n* (%)			
L1 ileal	16 (29.09)	46 (27.88)	1.000
L2 colonic	1 (1.82)	9 (5.45)	0.457
L3 ileocolonic	29 (52.73)	75 (45.45)	0.436
L4 upper	0 (0.00)	1 (0.61)	1.000
L4 + L1	4 (7.27)	13 (7.88)	1.000
L4 + L2	0 (0.00)	0 (0.00)	
L4 + L3	5 (9.09)	20 (12.12)	0.713
Montreal classification-behavior, n (%)			
B1 non-structuring, non-penetrating	29 (52.73)	91 (55.15)	0.876
B2 stricturing	14 (25.45)	48 (29.09)	0.729
B3 penetrating	4 (7.27)	13 (7.88)	1.000
B2 + B3	8 (14.55)	12 (7.27)	0.176
Perianal lesions, *n* (%)	36 (65.45)	113 (68.48)	0.803
MRI results of anal, *n* (%)			
No lesion	8 (14.55)	52 (31.52)	0.023*
Perianal inflammation	6 (10.91)	7 (4.24)	0.095
Anal fistula	27 (49.09)	97 (58.79)	0.272
Perianal abscess	14 (25.45)	39 (23.64)	0.927
Others	1 (1.82)	0 (0.00)	0.250
Gastrointestinal tumor, n (%)	0 (0.00)	1 (0.61)	1.000
Gastrointestinal bleeding, *n* (%)			
No	48 (87.27)	152 (92.12)	0.417
Once	6 (10.91)	8 (4.85)	0.120
Twice	0 (0.00)	3 (1.82)	0.575
3 times	0 (0.00)	1 (0.61)	1.000
> 3 times	1 (1.82)	1 (0.61)	0.438
Gastrointestinal perforation, *n* (%)			
No	47 (85.45)	156 (94.55)	0.040*
Once	8 (14.55)	9 (5.45)	0.040*
Twice	0 (0.00)	0 (0.00)	
3 times	0 (0.00)	0 (0.00)	
> 3 times	0 (0.00)	0 (0.00)	
Gastrointestinal obstruction, *n* (%)			
No	45 (81.82)	128 (77.58)	0.635
Once	10 (18.18)	24 (14.55)	0.667
Twice	0 (0.00)	8 (4.85)	0.206
3 times	0 (0.00)	1 (0.61)	1.000
> 3 times	0 (0.00)	4 (2.42)	0.574
Abdominal abscess, *n* (%)			
No	53 (96.36)	160 (96.97)	1.000
Once	2 (3.64)	5 (3.03)	1.000
Twice	0 (0.00)	0 (0.00)	
3 times	0 (0.00)	0 (0.00)	
> 3 times	0 (0.00)	0 (0.00)	
Intestinal fistula, *n* (%)			
No	53 (96.36)	154 (93.33)	0.526
Once	2 (3.64)	11 (6.67)	0.526
Twice	0 (0.00)	0 (0.00)	
3 times	0 (0.00)	0 (0.00)	
> 3 times	0 (0.00)	0 (0.00)	

*Statistically significant results

Abbreviations: *IQR* interquartile range, *MRI* magnetic resonance imaging

### Extraintestinal manifestations and surgical characteristics of familial CD and sporadic CD

Extraintestinal manifestations including peripheral arthritis/spondyloarthritis, pyoderma gangrenosum/aphthous ulcer/erythema nodosum/psoriasis, and iriditis/pigmentitis did not differ between familial CD and sporadic CD (Table 4).

**Table 4:** Extraintestinal manifestations of familial and sporadic Crohn’s disease (CD)

	Familial (*N* = 55)	Sporadic (*N* = 165)	*P* value
Extraintestinal manifestations, *n* (%)	13 (23.64)	46 (27.88)	0.660
Peripheral arthritis/spondyloarthritis, *n* (%)	6 (10.91)	18 (10.91)	1.000
Pyoderma gangrenosum/aphthous ulcer/erythema nodosum/psoriasis, *n* (%)	8 (14.55)	32 (19.39)	0.545
Iriditis/pigmentitis, *n* (%)	1 (1.82)	2 (1.21)	1.000

As for surgical characteristics, familial CD were more likely to have intestinal surgery history and have more times of intestinal surgery each patient. The number of intestinal surgeries was larger in familial CD (1.50[IQR 1.00–2.00] vs 1.00[IQR 1.00–2.00], *P* = 0.037) and history of intestinal surgery was more common in familial CD (50.91% vs 29.70%, *P* = 0.007). Besides, the proportion of patients who undergo surgery due to obstruction (32.73% vs 16.36%, *P* = 0.016), perforation (18.18% vs 7.27%, *P* = 0.038), and other reasons (12.73% vs 1.82%, *P* = 0.003) was significantly higher among the familial CD. Furthermore, familial CD had a higher percentage of the history of appendix surgery (38.18% vs 14.55%, *P* < 0.001) (Table 5).

**Table 5:** Surgical characteristics of familial and sporadic Crohn’s disease (CD)

	**Familial (*N* = 55)**	**Sporadic (*N* = 165)**	***P* value**
Age at the first intestinal surgery, years (median [IQR])	37.50 [31.50; 43.25]	36.00 [26.00; 49.00]	0.771
Resection of the intestine segments > 1 m, *n* (%)	7 (25.00)	4 (8.16)	0.086
Time between multiple surgeries, months (median [IQR])	59.00 [8.75; 105.75]	71.00 [14.25; 103.50]	0.318
Number of surgeries within 1 year of onset, *n* (%)	11 (39.29)	20 (40.82)	1.000
Number of surgeries within 2 years of onset, *n* (%)	12 (42.86)	26 (53.06)	0.532
Number of intestinal surgeries, *n* (median [IQR])	1.50 [1.00; 2.00]	1.00 [1.00; 1.00]	0.037*
History of intestinal surgery, *n* (%)	28 (50.91)	49 (29.70)	0.007*
Causes of intestinal surgery, *n* (%)			
Obstruction	18 (32.73)	27 (16.36)	0.016*
Perforation	10 (18.18)	12 (7.27)	0.038*
Intestinal fistula	7 (12.73)	17 (10.30)	0.803
Abdominal abscess	3 (5.45)	2 (1.21)	0.101
Canceration	1 (1.82)	0 (0.00)	0.250
Others	7 (12.73)	3 (1.82)	0.003*
Number of perianal surgeries, *n* (median [IQR])	1.00 [1.00; 2.00]	1.00 [1.00; 1.00]	0.067
History of perianal surgery, *n* (%)	21 (38.18)	54 (32.73)	0.565
Causes of perianal surgery, *n* (%)			
Anal fistula	13 (23.64)	32 (19.39)	0.629
Perianal abscess	15 (27.27)	33 (20.00)	0.346
Others	2 (3.64)	3 (1.82)	0.601
Number of endoscopic surgeries, *n* (median [IQR])	1.00 [1.00; 1.00]	1.00 [1.00; 1.00]	0.564
History of endoscopic surgery, *n* (%)	2 (3.64)	7 (4.24)	1.000
Causes of endoscopic surgery, *n* (%)			
Stenosis dilation	1 (1.82)	6 (3.64)	0.683
Canceration	0 (0.00)	0 (0.00)	
Others	1 (1.82)	1 (0.61)	0.438
History of appendix surgery, *n* (%)	21 (38.18)	24 (14.55)	< 0.001*

*Statistically significant results

Abbreviations: *IQR* interquartile range, *IBD* inflammatory bowel disease

### Follow-up of familial CD and sporadic CD

**Fig. 3:**
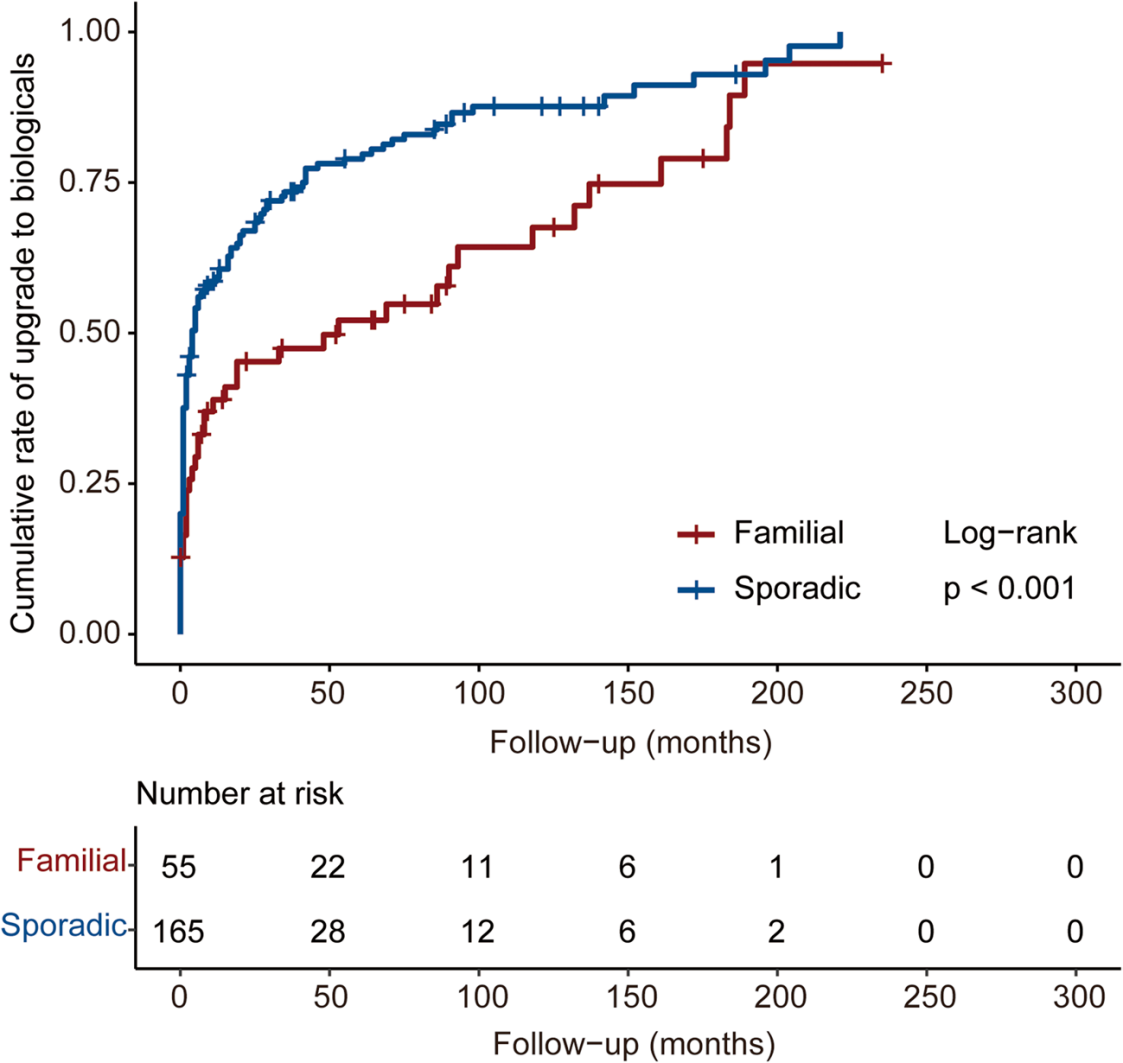
Cumulative rate of upgrade to biologicals in familial and sporadic Crohn’s disease (CD)

The median duration of follow-up in familial and sporadic CD patients was 54.00 months [IQR 21.50–125 months] and 41.00 months [IQR 14.00–79.00 months], respectively (*P* = 0.017). For the current maintenance drug, the percentage of patients taking 5-aminosalicylic (5-ASA) was significantly higher among the familial CD (9.09% vs 0.61%, *P* = 0.004), while the percentage of patients taking biologicals was significantly lower among the familial CD (61.82% vs 76.97%, *P* = 0.043). During follow-up, the familial CD was associated with a lower tendency to upgrade to biologicals (69.09% vs 85.37%, *P* = 0.013) (Table 6). The cumulative rate of upgrade to biologicals was also lower in familial CD (*P* < 0.001) (Fig. 3). During follow-up, the familial CD was associated with an increased possibility of experiencing gastrointestinal obstruction (23.64% vs 11.52%, *P* = 0.047) and abdominal abscess (9.09% vs 2.42%, *P* = 0.045). For current disease behavior, familial CD had a higher percentage of structuring and penetrating behavior (29.09% vs 13.94%, *P* = 0.019) (Table 6).

**Table 6:** Follow-up of familial and sporadic Crohn’s disease (CD)

	**Familial (*N* = 55)**	**Sporadic (*N* = 165)**	***P* value**
Duration of follow-up, *n* (median [IQR])	54.00 [21.50;125.00]	41.00 [14.00;79.00]	0.017*
Current maintenance drug, *n* (%)			
No	6 (10.91)	14 (8.48)	0.787
5-ASA	5 (9.09)	1 (0.61)	0.004*
Steroid	2 (3.64)	3 (1.82)	0.601
Immunosuppressant	15 (27.27)	26 (15.76)	0.089
Biologicals	34 (61.82)	127 (76.97)	0.043*
Enteral nutrition	3 (5.45)	5 (3.03)	0.416
Clinical trial	0 (0.00)	2 (1.21)	1.000
Upgrade to biologicals, *n* (%)	38 (69.09)	140 (85.37)	0.013*
Type of biologicals, *n* (%)			
Infliximab	33 (60.00)	121 (73.33)	0.089
Adalimumab	1 (1.82)	4 (2.42)	1.000
Vedolizumab	1 (1.82)	2 (1.21)	1.000
Ustekinumab	3 (5.45)	10 (6.06)	1.000
Risankizumab	0 (0.00)	1 (0.61)	1.000
Clinical trial	0 (0.00)	2 (1.21)	1.000
Loss of response to biologicals, *n* (%)	7 (18.42)	14 (10.22)	0.169
Current disease location, *n* (%)			
L1 ileal	15 (27.27)	45 (27.27)	1.000
L2 colonic	1 (1.82)	9 (5.45)	0.457
L3 ileocolonic	29 (52.73)	74 (44.85)	0.391
L4 upper	0 (0.00)	1 (0.61)	1.000
L4 + L1	4 (7.27)	12 (7.27)	1.000
L4 + L2	0 (0.00)	0 (0.00)	
L4 + L3	6 (10.91)	24 (14.55)	0.650
Disease location change, *n* (%)	2 (3.64)	3 (1.82)	0.601
Current disease behavior, *n* (%)			
B1 non-stricturing, non-penetrating	19 (34.55)	75 (45.45)	0.208
B2 stricturing	14 (25.45)	52 (31.52)	0.497
B3 penetrating	6 (10.91)	15 (9.09)	0.895
B2 + B3	16 (29.09)	23 (13.94)	0.019*
Disease behavior progression, *n* (%)	15 (27.27)	25 (15.15)	0.069
Gastrointestinal tumor, *n* (%)	1 (1.82)	0 (0.00)	0.250
Gastrointestinal bleeding, *n* (%)			
No	52 (94.55)	158 (95.76)	0.714
Once	3 (5.45)	2 (1.21)	0.101
Twice	0 (0.00)	2 (1.21)	1.000
3 times	0 (0.00)	1 (0.61)	1.000
> 3 times	0 (0.00)	2 (1.21)	1.000
Gastrointestinal perforation, *n* (%)			
No	51 (92.73)	161 (97.58)	0.110
Once	4 (7.27)	4 (2.42)	0.110
Twice	0 (0.00)	0 (0.00)	
3 times	0 (0.00)	0 (0.00)	
> 3 times	0 (0.00)	0 (0.00)	
Gastrointestinal obstruction, *n* (%)			
No	42 (76.36)	146 (88.48)	0.047*
Once	9 (16.36)	12 (7.27)	0.085
Twice	1 (1.82)	2 (1.21)	1.000
3 times	1 (1.82)	4 (2.42)	1.000
> 3 times	2 (3.64)	1 (0.61)	0.155
Abdominal abscess, *n* (%)			
No	50 (90.91)	161 (97.58)	0.045*
Once	5 (9.09)	4 (2.42)	0.045*
Twice	0 (0.00)	0 (0.00)	
3 times	0 (0.00)	0 (0.00)	
> 3 times	0 (0.00)	0 (0.00)	
Intestinal fistula, *n* (%)			
No	46 (83.64)	149 (90.30)	0.270
Once	8 (14.55)	16 (9.70)	0.454
Twice	1 (1.82)	0 (0.00)	0.250
3 times	0 (0.00)	0 (0.00)	
> 3 times	0 (0.00)	0 (0.00)	

*Statistically significant results

Abbreviations: *IQR* interquartile range, *5-ASA* 5-aminosalicylic acid

### Phenotypic concordance among 43 pairs of familial CD

Twenty-nine out of the 43 pairs (67.44%) of familial CD were concordant for age at onset ≤ 40 years old. Concordance of disease location, disease behavior, and perianal lesions at diagnosis was 60.47%, 44.19%, and 60.47%, respectively. Upgrading to biologicals occurred in 25 of the 43 pairs (58.14%). For current disease location and behavior, concordance was 55.81% and 27.91%. The concordance was 51.16% for the history of intestinal surgery (Table 7).

**Table 7:** Phenotypic concordance among 43 pairs of familial Crohn’s disease (CD)

	**Concordant pairs**	**Concordance (%)**
Age at onset ≤ 40 years old	29	67.44
Disease location at diagnosis	26	60.47
Disease behavior at diagnosis	19	44.19
Perianal lesions at diagnosis	26	60.47
Upgrade to biologicals	25	58.14
Current disease location	24	55.81
Current disease behavior	12	27.91
History of intestinal surgery	22	51.16

## Discussion

This study describes the prevalence, demographic characteristics, clinical characteristics, extraintestinal manifestations, surgical characteristics, follow-up, and phenotypic concordance of familial CD and sporadic CD. The results indicated that familial CD was associated with a more aggressive clinical phenotype, such as more intestinal perforation at onset, more MRI results of the anal lesion at diagnosis, more likelihood of intestinal surgery history, more times of intestinal surgery, and more gastrointestinal obstruction and abdominal abscess during follow-up.

In terms of age, previous studies have found that familial IBD cases have an earlier age of onset and diagnosis [4, 22, 23]; however, this study did not find significant differences in age of onset or diagnosis between familial and sporadic cases, which may be due to the small sample size.

As for extraintestinal manifestations, there was no difference between familial and sporadic CD in our study, which is similar to the findings of Saberzadeh-Ardestani et al. and Chung et al. [7, 15]. However, in other studies, familial IBD was associated with 1.8–5.6 times higher extraintestinal manifestations [24, 25]. This variation may be attributed to the genetic and environmental diversity among different geographic areas.

Our major outcome is that familial CD shows a more aggressive clinical phenotype and needs more surgery. However, the literature about the severity of familial CD is controversial. Previous studies found no effect of family history on disease complications and need for surgery, including a small study of 17 Korean familial CD [15], a study of 181 Jewish CD [26], a cohort of Norway patients [27], a cohort of Finnish patients [28], and a large cohort of 240 Iranian familial CD [7]. In contrast to these findings and consistent with our observations, several studies have shown that familial CD has a higher proportion of perianal disease and penetrating behavior [22], is more aggressive [6], and has a higher prevalence of fistulizing disease [5]. This is in accordance with our finding as we found that familial CD had more intestinal perforations at onset, more MRI results of anal lesion at diagnosis, and more gastrointestinal perforation at diagnosis. As for surgical characteristics, we found that familial CD were more likely to have intestinal surgery history, have more times of intestinal surgery, and have more likelihood to undergo surgery due to obstruction and perforation, which correspond to a more aggressive clinical phenotype.

In terms of follow-up, this study found that the median follow-up time was significantly longer in familial CD than in sporadic CD. One possible explanation is that familial CD manifests as a phenotypically aggressive condition, necessitating regular follow-ups and intensive medical care, which consequently leads to better treatment adherence. As for maintenance drugs, most studies indicated that the rate of biological use was higher in familial CD [5, 23, 29], and some studies showed no difference [4, 30]. However, in this study, familial CD patients were associated with lower rates of escalation to biologicals and current biologicals use for maintenance of remission compared with sporadic CD. One possible reason is that familial CD patients have better treatment willingness and compliance, receive clinical intervention earlier, and achieve better disease control, thus less requiring biologicals. Another potential explanation is that familial IBD patients have higher surgery rates, and after surgical resection resulting in disease control, they did not need biologicals for maintenance but rather maintained remission using traditional 5-ASA and immunosuppressants instead. On the other hand, the affordability issues surrounding biologics, coupled with the easy availability and lower cost of immunomodulators in this context, could result in the use of less advanced therapies for familial CD.

Although our study demonstrated several differences in clinical phenotype between familial CD and sporadic CD, some limitations remain to be addressed. First, this study was mainly done at an IBD center of a tertiary hospital. The disease conditions tend to be more complex and severe, leading to selection bias that may not reflect the real study population. Second, this study was performed using the hospital-based registry and was not a population-based cohort study. Therefore, a population-based cohort study will be needed in the future. Third, the small sample size, short follow-up period, and lack of inclusion of more objective data such as laboratory tests, imaging, endoscopy, and pathology, reduce the reliability of the results. Larger and multi-center cohorts are essential to more robustly define the similarities and differences between familial and sporadic IBD. In addition, this study did not investigate genetic aspects of familial CD, which could provide crucial insights into inheritance patterns and disease mechanisms.

In conclusion, this study provides evidence of a more aggressive clinical phenotype in patients with familial CD compared with sporadic CD. These findings have important implications for clinical practice and would help the clinician to predict the course of the disease, stratify familial IBD as high risk, and initiate treatment accordingly.

## Data Availability

Data will be made available on request.
